# Efficacy of simulation-based training for airway management in preparing hospitals for the COVID-19 pandemic: a systematic review

**DOI:** 10.3389/fmed.2025.1656737

**Published:** 2025-12-09

**Authors:** Myrtha Magdalena Kohler, Michaela Kolbe, Benedict Körtgen, Stephanie Angst, Anna Maria Stephanie Barbul, Lisa Seufert, Radek Hasal, Lea Bührer, Ulrike Held, Bastian Grande

**Affiliations:** 1 Institute for Anesthesiology and Perioperative Medicine, University Hospital Zurich, Zurich, Switzerland; 2 Simulation Center, University Hospital Zurich, Zurich, Switzerland; 3 Department of Health Sciences and Technology, ETH Zurich, Zurich, Switzerland; 4 Institute of Anesthesiology, Seespital Horgen, Horgen, Switzerland; 5 Institute of Intensive Care Medicine, University Hospital Zurich, Zurich, Switzerland; 6 Institute of Anesthesiology, Kantonsspital Winterthur, Winterthur, Switzerland; 7 Department of Anesthesia, Sir Charles Gairdner Hospital, Perth, WA, Australia; 8 Institute of Anesthesiology, Stadtspital Zurich, Zurich, Switzerland; 9 Epidemiology, Biostatistics and Prevention Institute, University of Zurich, Zurich, Switzerland

**Keywords:** simulation training, airway management, difficult intubation, COVID, pandemic

## Abstract

**Background:**

In response to the coronavirus pandemic, hospitals worldwide implemented simulation-based training to help healthcare providers (HCPs) adapt to revised protocols for airway management in patients with infectious coronavirus disease 2019 (COVID-19). We conducted a systematic review of simulation-based studies on airway management in COVID-19 patients, with the aim of analyzing the findings of these studies and consolidating evidence-based recommendations to optimize responses to possible future pandemics.

**Methods:**

We performed a systematic literature search of PubMed, Embase, Medline, and the Cochrane Library on 25 August 2022. As different studies measured different outcomes (e.g., only confidence, only knowledge, or both) in different ways, a random-effects model was used for meta-analysis and change scores were calculated.

**Results:**

The systematic review included 20 studies after screening 141 articles. The meta-analysis revealed significant improvements in participants' confidence and knowledge after simulation training, as evidenced by negative standardized mean differences (SMDs, Cohen's d). Sensitivity analysis confirmed that the results were robust across various correlation estimates. However, there was a high risk of publication bias, as funnel plots showed asymmetry and studies fell outside the 95% confidence interval.

**Conclusion:**

This systematic review highlights the effectiveness of simulation training in improving healthcare providers' confidence and knowledge regarding airway management during pandemics. The findings underscore the positive impact of simulation-based education, as demonstrated by significant improvements from pre-training to post-training assessments. However, the observed publication bias suggests that additional high-quality, unbiased studies are necessary to strengthen the evidence base and inform future training programs for pandemic preparedness.

**Systematic review registration:**

PROSPERO, CRD42022293708.

## 1 Introduction

Due to the rapid spread of coronavirus disease 2019 (COVID-19) beyond China, hospitals worldwide were forced to prepare for an impending pandemic in a very short period of time. Recent literature has also emphasized the concept of the physiologically difficult airway and the importance of associated guidelines in the context of COVID-19. Moreover, airway management under pandemic conditions is associated with additional stress among healthcare providers (HCPs), the need to limit the number of team members to reduce exposure, and the challenges of working with personal protective equipment (PPE).

The increased risk of virus transmission to healthcare providers (HCPs) was one of the biggest challenges for hospitals and HCPs (1, 2). Airway manipulations are high-risk aerosol-generating procedures (AGPs), representing one of the greatest risks of transmission in case of a respiratory virus (3, 4). Therefore, normal airway algorithms were adapted by different airway and anesthesiologic societies to make them more secure for patients and the medical staff performing these procedures (5, 6).

The viral load in the sputum and upper and lower airway secretions of COVID-19 patients has been shown to be particularly high (7), making the transmission of COVID-19 from patients to HCPs during airway manipulations more probable (2–4, 8). In addition, due to the exposure to a higher viral load, case fatality rates have been found to be significantly higher among HCPs compared to patients with community-acquired COVID-19 (9). Similarly, the risk arising from respiratory viruses to HCPs was apparent during the severe acute respiratory syndrome (SARS) epidemic in 2003, with 21% of all infected patients being HCPs (10).

However, HCPs are not the only individuals at risk. COVID-19 patients who are critically ill and need emergency intubation face a higher risk of hypoxemia and even cardiac arrest compared to those receiving standard intubation (5, 11).

The algorithms published by airway and anesthesiologic societies (5, 6) recommended good preoxygenation, rapid sequence induction, avoidance of bag-mask ventilation (if necessary, two-handed, two-person technique), and intubation by the most experienced HCP using a video laryngoscope (5). This approach maximized first-pass success and minimized aerosol-generating procedures (AGPs).

After the algorithms were adapted, it was important that HCPs knew about the alterations and were able to perform safe intubation in COVID-19 patients.

Therefore, Cook et al. (5) recommended conducting simulation training in their consensus guidelines to implement protocols quickly and efficiently and to identify local problems prior to real-patient situations, as it had been done in previous epidemics (12). At the beginning of the pandemic, hospitals worldwide employed various simulation-based training approaches to train HCPs on the correct donning and doffing of personal protective equipment (PPE) and to ensure safe intubation with minimal AGPs. However, to date, there is no systematic overview of how simulation-based training can be used effectively to teach airway management for suspected and confirmed COVID-19 patients.

As the COVID-19 pandemic was a worldwide medical crisis that lasted several years and we still do not know if there will be a higher pathogenic mutation in the future (13–15), it is crucial to provide HCPs with safe airway management training (4, 16). Therefore, the aim of this systematic review is to synthesize evidence on the efficacy of simulation-based training for airway management in the COVID-19 context, with particular focus on its impact on provider competence, team preparedness, and safety outcomes.

We are convinced that we can learn important lessons regarding preparedness and protecting HCPs in anesthesiology, intensive care, and emergency medicine for possible future pandemics by analyzing our performances during the COVID-19 pandemic.

## 2 Methods

The protocol for this study was registered on the International Prospective Register of Systematic Reviews (PROSPERO) of the National Institute for Health Research (NIHR) and published under registration ID CRD42022293708. The review was conducted according to the Preferred Reporting Items for Systematic Reviews and Meta-Analyses (PRISMA) guidelines.

### 2.1 Eligibility criteria

We included all published studies in which HCPs had to perform oral intubation on COVID-19 patients during simulation training. No restrictions were placed on study design. The co-primary outcomes were the perceived usefulness of the simulation for pandemic preparedness, confidence gained through the simulation, and improved preparedness to treat COVID-19 patients. We excluded studies that examined (a) contamination in simulation scenarios with different devices, mostly aerosol boxes; (b) non-oral airway procedures (mostly tracheostomy); (c) no simulation of airway scenarios, only providing algorithms or simulation scenarios; (d) ventilator-related issues; (e) single case reports; (f) conference abstracts or posters; and (g) lists of airway equipment for COVID-19 patients.

No language restrictions were applied in the search. We did not include gray literature. The last search was conducted on 25th August 2022. The inclusion criteria focused on studies evaluating simulation-based training for airway management in the context of COVID-19, while exclusion criteria included studies without primary data or those not addressing simulation in this context.

### 2.2 Search strategy

On 25th August 2022, we conducted a search on PubMed, Medline, the Cochrane Library, and Embase using the keywords “airway,” “management,” “COVID,” and “simulation.” We excluded publications containing the keyword “box,” as there have been many publications investigating the use of aerosol boxes for intubation of COVID-19 patients. We refrained from applying any other limitations, especially language restrictions or time cutoffs. The search algorithm for each database can be found in Supplementary Digital Content 1. The aim was to find articles that compared and assessed simulation as a training method to prepare HCPs for the COVID-19 pandemic. We aimed to compare studies in which simulation training for airway management in COVID-19 patients was performed. We could not find a review article or meta-analysis on this important subject at the time of our search.

### 2.3 Selection process

Study selection was conducted by two reviewers, MK and BK, who independently screened the titles and abstracts of all publications identified during the literature search. In case of insufficient information in the abstract, the publications were included for full-text review. Disagreements were resolved by consensus. During the full-text review, eligibility was again independently assessed by both reviewers. Disagreements were resolved through arbitration by a third reviewer, BG.

### 2.4 Data collection process and data items

Data collection was conducted independently by four reviewers. The first round was completed by MK, and the second round was divided among SA, AB, and LS using a customized Excel database. In case of discrepancies in data extraction, discussion was used to resolve them. Any measure of effect related to simulation training was eligible for inclusion. We categorized outcomes according to the four levels of the Kirkpatrick model—reaction, learning, behavior, and results—to provide a structured framework for interpretation (17). Level 1 represents reaction (e.g., the satisfaction or confidence of the participants), Level 2 reflects learning (e.g., acquisition of knowledge and skills), Level 3 assesses changes in behavior (application of the acquired knowledge or skills at work), and Level 4 measures results (better patient outcomes at work due to the training). Applying the Kirkpatrick model allowed us to contextualize improvements in knowledge and confidence as indicators of both learning and behavioral changes, supporting the robustness of our conclusions.

No restrictions were imposed regarding the number of participants in the included studies.

### 2.5 Study risk of bias assessment

To assess the risk of bias, we used the Scottish Intercollegiate Guidelines Network (SIGN) checklist for cohort studies (18). Most well-known checklists for assessing risk of bias are designed for studies with control and intervention groups. Due to their emphasis on blinding and differences between these groups, they were not suitable for the design of the included simulation studies. All reviewed publications had only one group of participants, with data regarding their knowledge or confidence collected before and after the simulation training. Ratings were assigned independently by two reviewers (MK and AB), and discrepancies were resolved by consensus. A graphic overview was created to provide an outline of the study quality.

### 2.6 Effect measures

Two outcomes were investigated: The level of comfort, which was captured as confidence in the data set, and the level of knowledge after the simulation-based training.

Some parameters necessary for meta-analysis were not reported directly in the publication but could be retrieved from other information that was reported in the publication. In these situations, the Wald confidence interval was used, along with the approximation of the standard deviation (SD) and mean as described by Wan et al. (19).

Descriptive statistics, stratified by publication year, are reported as follows: Mean and standard deviation (SD) for continuous variables, median and interquartile range (IQR) for ordinal or skewed continuous variables, and frequencies and percentages for categorical variables.

### 2.7 Synthesis methods

As the different studies measured different outcomes (e.g., only confidence, only knowledge, or both) in different ways (5-point Likert scale, 7-point Likert scale, Multiple Choice Questions), we had a large amount of missing data. For the meta-analysis, a random-effects model was employed due to the expected substantial between-study heterogeneity in the meta-analysis of observational studies. The meta-analysis was performed using the function rma() from the package metafor. As the studies applied different scales of measurement for the primary outcomes, no direct overall effect measure could be estimated. However, forest plots were generated to provide a visual representation of the primary outcomes. Change scores were calculated for the studies to be able to compute a random effects model, as well as standardized mean differences (SMDs) for paired data. A correlation coefficient ρ of 0.5 was assumed for these calculations. A random effects model was used for meta-analysis. The heterogeneity parameter τ^2^ was computed by default using the restricted maximum likelihood (REML) estimator for the random effects models. The number of studies in the meta-analyses was low; therefore, meta-regression could not be performed to evaluate sources of heterogeneity. To do this, a minimum of 10 studies would be necessary. To still evaluate sources of heterogeneity, graphical assessments of possible sources of heterogeneity were performed.

*I*^2^ and χ^2^ are reported. The χ^2^ test assesses the significance of heterogeneity but does not provide measurements. An index of heterogeneity is the *I*^2^ by Higgins, see Bland (20), chapter 17.5. The *I*^2^ is the percentage of the χ^2^ statistic that is not explained by the variation within the studies. An *I*^2^ value from 0% to 40% might not be important, a value from 30% to 60% may represent moderate heterogeneity, a value from 50% to 90% may represent substantial heterogeneity, and a value from 75% to 100 % indicates considerable heterogeneity. Funnel plots were used to assess possible publication bias.

For all analyses, a nominal level (α*)* of 5% was used. Accordingly, the default confidence interval width was set to 95%.

As different measurement types (e.g., 5-point Likert scales, numeric rating scales from 0 to 10, surveys without scales, 7-point scales) were used to report the confidence and knowledge gained through training, it was not possible to derive overall effects from the given data. Therefore, the change score between the post- and pre-simulation results was derived. Consequently, the data were to some degree standardized as the differences were being investigated and not the raw scores. However, as the scores did not have the same range (from 2 to 15 points possible), it was not possible to completely standardize the values in this way.

The mean change in each group was calculated by subtracting the post-simulation mean from the baseline mean, see the following formula:


$$\begin{array}{l} Change\;Score=\;{\bar{x}}_{pre-sim}-\;{\bar{x}}_{post-sim} \end{array}$$

The SD of a change score was computed in the following way [Higgins et al. (21), chapter 6.5.2.8].


$$\begin{array}{l} SD_{change}=\sqrt{SD_{\;pre-sim}^{2}+\;SD_{\;post-sim}^{2}\;-\;(2\;*\rho^{*}SD_{pre-sim}\;*SD_{post-sim})} \end{array}$$

Where ρ = 0.5 was set by definition.

### 2.8 Sensitivity analysis

The correlation parameter ρ was changed in a sensitivity analysis to quantify the effect of the choice on the results. Moreover, the estimation method for τ^2^ was changed to the DerSimonian–Laird (DL) estimator in a sensitivity analysis. If the results based on the sensitivity analysis remained unchanged, they were considered robust.

All analyses were performed using the R programming language (22) in combination with dynamic programming with knitr in a fully scripted way.

## 3 Results

### 3.1 Study selection

In our search of the electronic databases, we found 141 articles after removing duplicates, of which 32 met the inclusion criteria. Inter-reviewer reliability indicated substantial agreement and was calculated using means of Cohen's Kappa (κ = 0.72; *n* = 141 articles, 129 agreements and 12 disagreements).

After the full-text review, we excluded 13 publications because they did not meet the inclusion criteria after all or were posters from conferences or letters to the editor. In addition, we searched the reference lists of the included articles to find other relevant publications. In this way, we found another article that met the inclusion criteria. Therefore, in total, we found 20 articles that met our inclusion criteria, as shown in our PRISMA flow chart (see Supplementary Digital Content 2).

### 3.2 Study characteristics

A list of the screened publications, including the 20 studies that met our inclusion criteria, can be found in Supplementary Digital Content 3.

### 3.3 Risk of bias in the studies

We screened the risk of bias according to the SIGN checklist for cohort studies (18). For ease of visualization, we created a graphic diagram. The following tables show our assessment process and our results according to the checklist's questions (Table 1):

**Table 1:** Risk of bias assessment—questions.

**Question number**	**Checklist question**	Low quality/high risk of bias 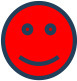	High quality 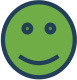	Acceptable quality 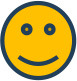	Uncertain/cannot be determined 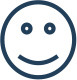
1	The study addresses an appropriate and clearly focused question	No	Yes	Can't say	
2	The Study indicates how many of the people asked to take part did so	No	Yes		Does not apply
3	The likelihood that some eligible subjects might have the outcome at the time of enrolment is assessed and taken into account in the analysis	No	Yes	Can't say	Does not apply
4	Comparison is made between full participants and those lost to follow up, by exposure status	No	Yes	Can't say	Does not apply
5	The outcomes are clearly defined	No	Yes	Can't say	
6	The assessment of outcome is made blind to exposure status. If the study is retrospective this may not be applicable	No	Yes	Can't say	Does not apply
7	The method of assessment of exposure is reliable	No	Yes	Can't say	
8	Evidence from other sources is used to demonstrate that the method of outcome assessment is valid and reliable	No	Yes	Can't say	Does not apply
9	Exposure level or prognostic factor is assessed more than once	No	Yes	Can't say	Does not apply
10	The main potential confounders are identified and taken into account in the design and analysis	No	Yes	Can't say	
11	Have confidence intervals been provided?	No	Yes		
12	How well was the study done to minimize the risk of bias or confounding?	Unacceptable—reject	High quality	Acceptable	
13	Taking into account clinical considerations, your evaluation of the methodology used, and the statistical power of the study, do you think there is clear evidence of an association between exposure and outcome?	No	Yes	Can't say	
14	Are the results of this study directly applicable to the patient group targeted in this guideline?	No	Yes		

The complete checklist included questions that did not apply to any of our studies due to their study design. The cohort studies checklist was the best fit, but as we did not compare two different groups, the questions regarding homogeneity or dropouts from each arm of the study could not be answered. In addition, there was no blinding, as the participants knew about the simulation training and the aim of the training. To make our table clearer and for ease of visualization, we excluded the following questions. The answer to all of them would have been “does not apply” in all of our included studies.

1. The two groups being studied are selected from source populations that are comparable in all respects other than the factor under investigation.
2. What percentage of individuals or clusters recruited into each arm of the study dropped out before the study was completed?
3. Where blinding was not possible, there is some recognition that knowledge of exposure status could have influenced the assessment of outcome.

Among the included studies, the majority were rated as moderate quality, with fewer studies rated as high or low quality; a summary distribution is provided in Table 2.

**Table 2:**
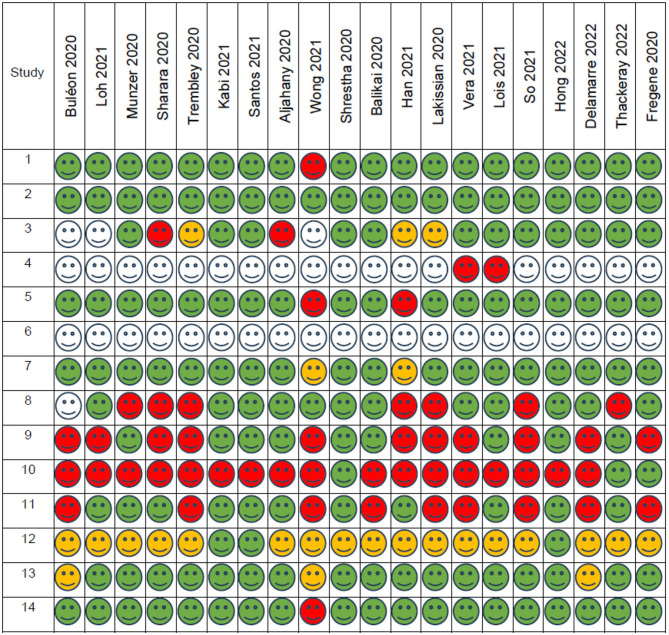
Risk of bias assessment.

### 3.4 Results of the individual studies

Of all 20 studies, five provided numeric data on the confidence of the participants before and after the simulation training. In addition, four studies provided data on knowledge before and after the training. The remaining studies provided only post-training data, so we could not compare changes in knowledge or confidence, or did not provide numeric data at all.

Across the studies, “confidence” referred to various aspects, including self-confidence in technical airway skills, team performance, handling of PPE, and stress management. When different data were provided regarding the confidence of various skills, we focused on confidence related to airway management.

**Figure 1:**
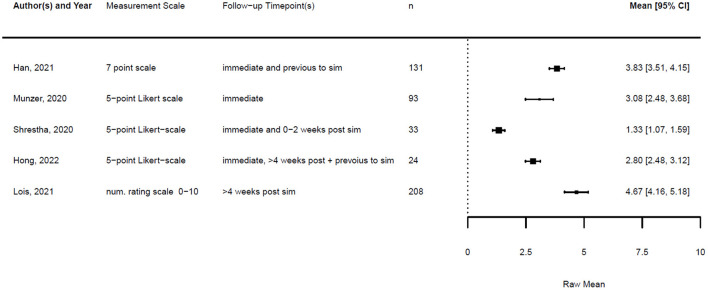
Forest plot for confidence pre simulation (*n* = 5). Reported means are on original scale used in the study.

**Figure 2:**
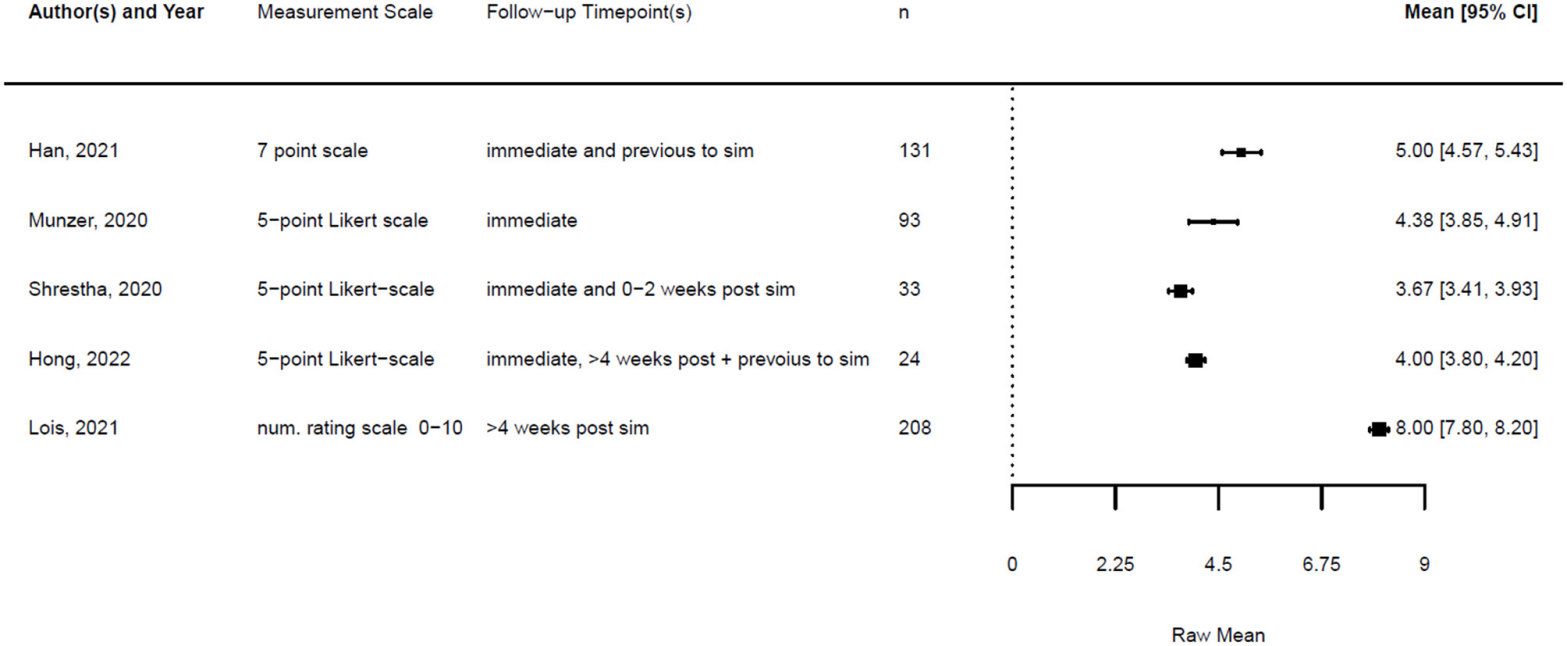
Forest plot for confidence post simulation (*n* = 5). Reported means are on original scale used in the study.

**Figure 3:**
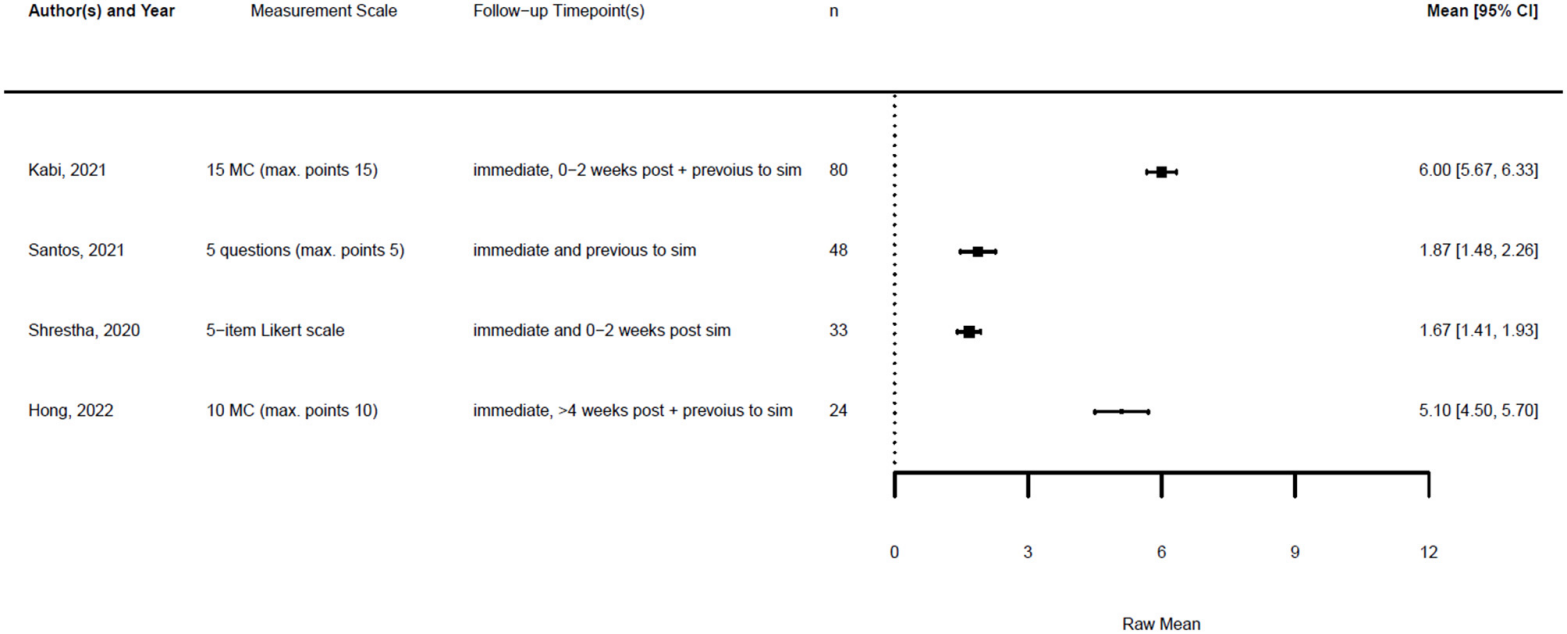
Forest plot for knowledge pre simulation (*n* = 4). Reported means are on original scale used in the study.

**Figure 4:**
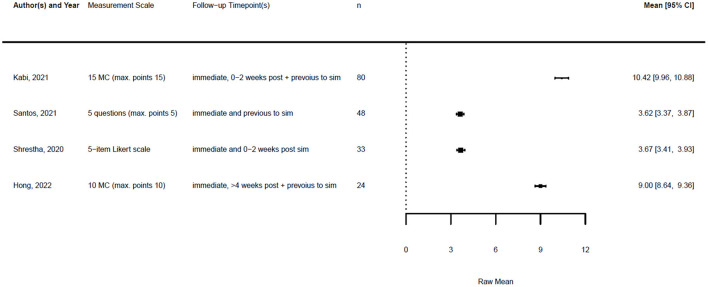
Forest plot for knowledge post simulation (*n* = 4). Reported means are on original scale used in the study.

**Figure 5:**
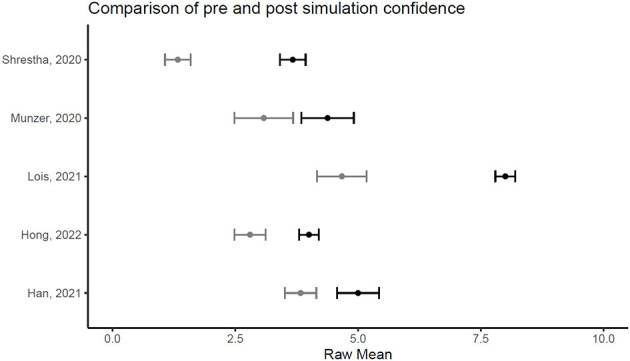
Forest plot for confidence comparing pre and post simulation per paper (*n* = 5). Grey indicates the pre simulation confidence reported, black the post confidence. Reported means are on original scale used in the study.

**Figure 6:**
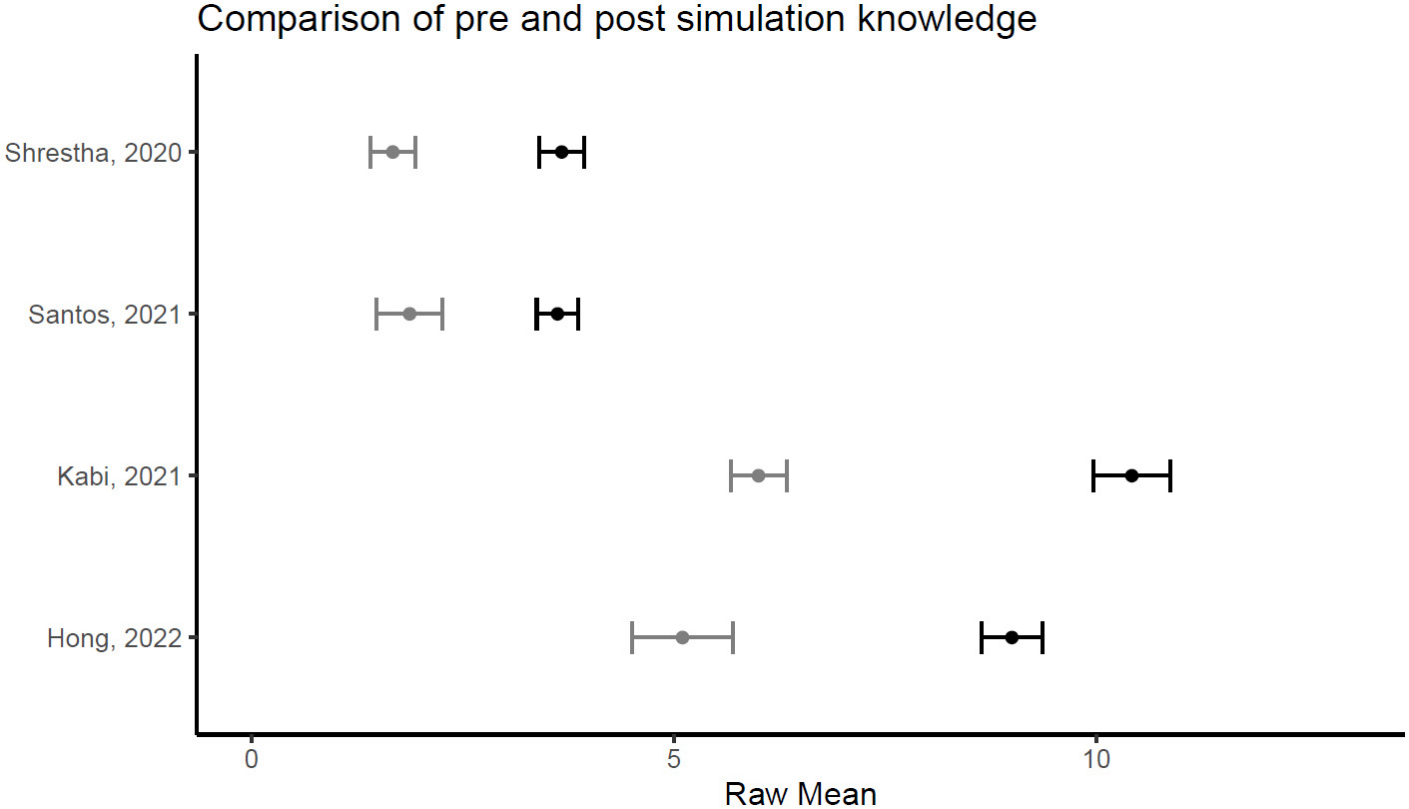
Forest plot for knowledge comparing pre and post simulation per paper (*n* = 5). Grey indicates the pre simulation confidence reported, black the post confidence. Reported means are on original scale used in the study.

Figures 1, 2 illustrate the mean confidence pre- and post-simulation, including the 95% confidence interval. Pre- and post-training knowledge scores are provided in Figures 3, 4. Figures 5, 6 compare the means and 95% confidence intervals before and after the simulation for the two primary outcomes, respectively.

### 3.5 Results of the synthesis method

**Figure 7:**
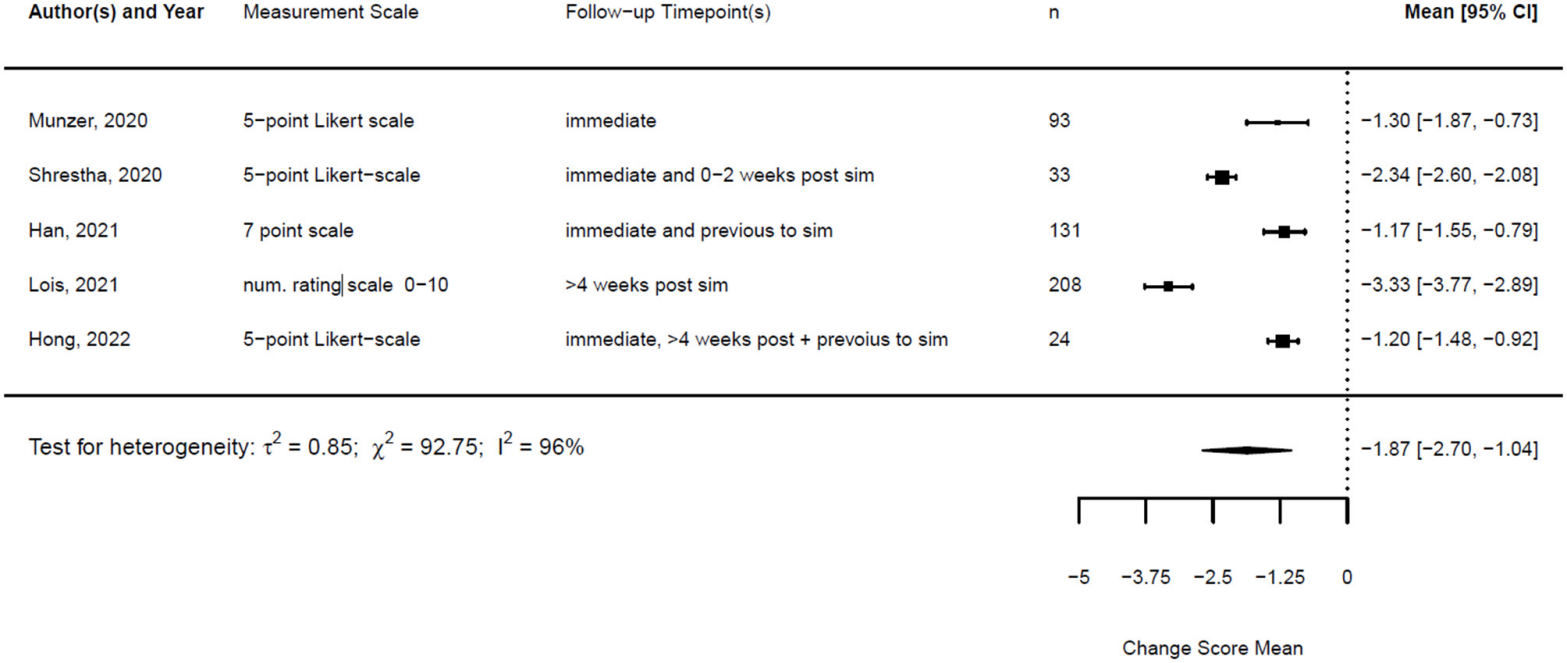
Forest plot for change scores of confidence (*n* = 5).

**Figure 8:**
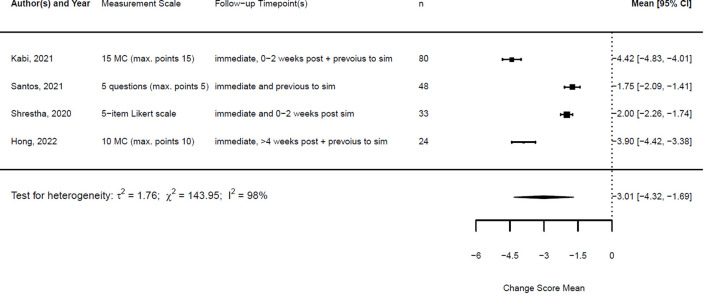
Forest plot for change scores of knowledge (*n* = 4).

As described in the methods, the studies were heterogeneous; therefore, we compared the change score of the different studies. The studies and the results of the meta-analyses are illustrated in Figures 7, 8. As the observed post-simulation confidence and knowledge means were subtracted from the baseline before training, a score less than zero indicated a positive training effect. A score of zero demonstrated no training effect, while a value greater than zero indicated a negative training effect, meaning reduced confidence or knowledge after the simulation.

The standardized mean difference (SMD) of a paired sample is also called Cohen's d, and together with its standard error, it can be derived from the given data. This is implemented in the metafor package in R (23). Therefore, a random effects model was fitted for both outcomes using the standardized mean difference as the effect measure.

The formula for the calculation of the SMD reads is as follows:


$$\begin{array}{l} SMD=\frac{{\bar{x}}_{pre-sim}-\;{\bar{x}}_{post-sim}\;}{SD} \end{array}$$

**Figure 9:**
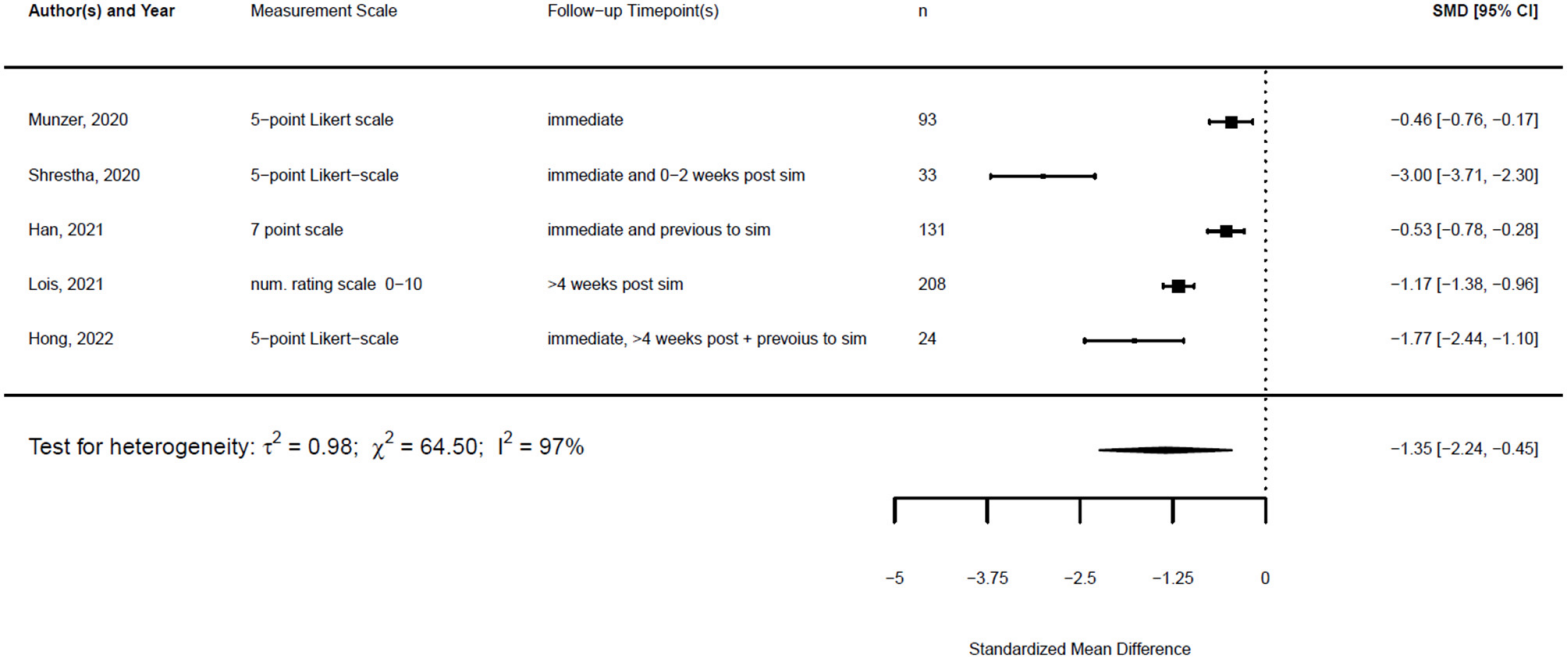
Forest plot for standardized mean difference of confidence (*n* = 5).

**Figure 10:**
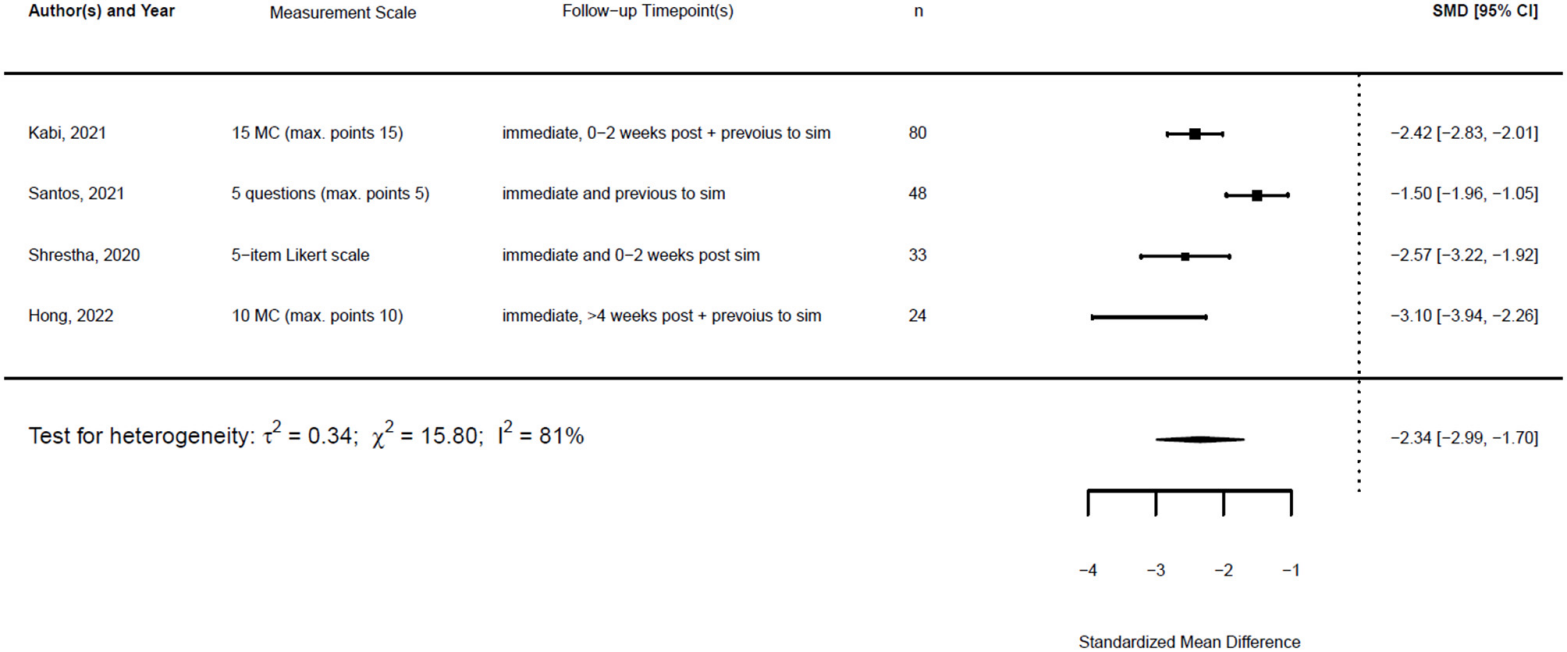
Forest plot for standardized mean difference of knowledge (*n* = 4).

With the given formula, a negative effect was expected. The post-simulation results were subtracted from the pre-simulation results. A value equal to zero indicated no effect, while a value greater than zero indicated a reduction in confidence or knowledge following the simulation/training. A negative effect indicated a training effect, as confidence or knowledge after training was higher than before (see Figures 9, 10).

### 3.6 Sensitivity analysis

**Figure 11:**
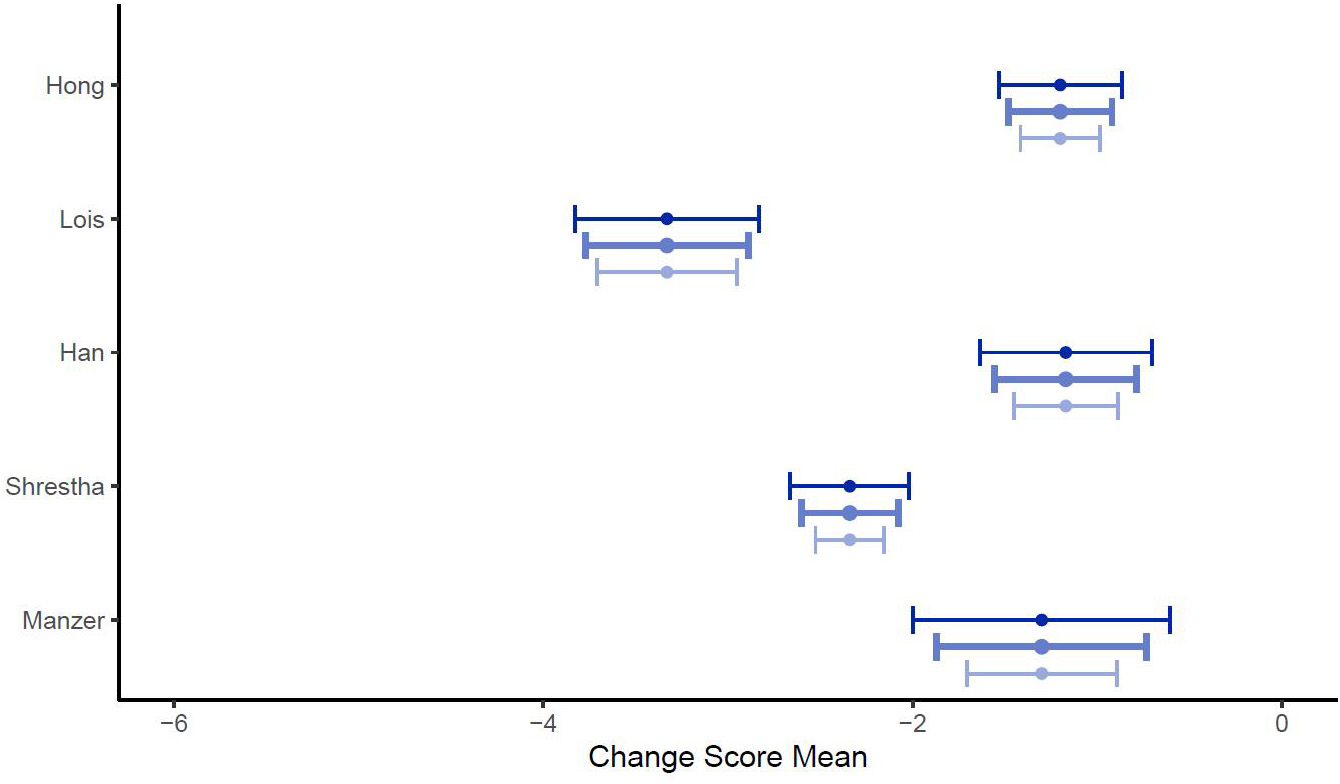
Forest plot for change scores of confidence (*n* = 5) for ρ = (0.25, 0.5, 0.75) to calculate the standard deviation of the change score. Medium blue indicates ρ = 0.5, dark blue stands for ρ = 0.25 and light blue for ρ = 0.75.

**Figure 12:**
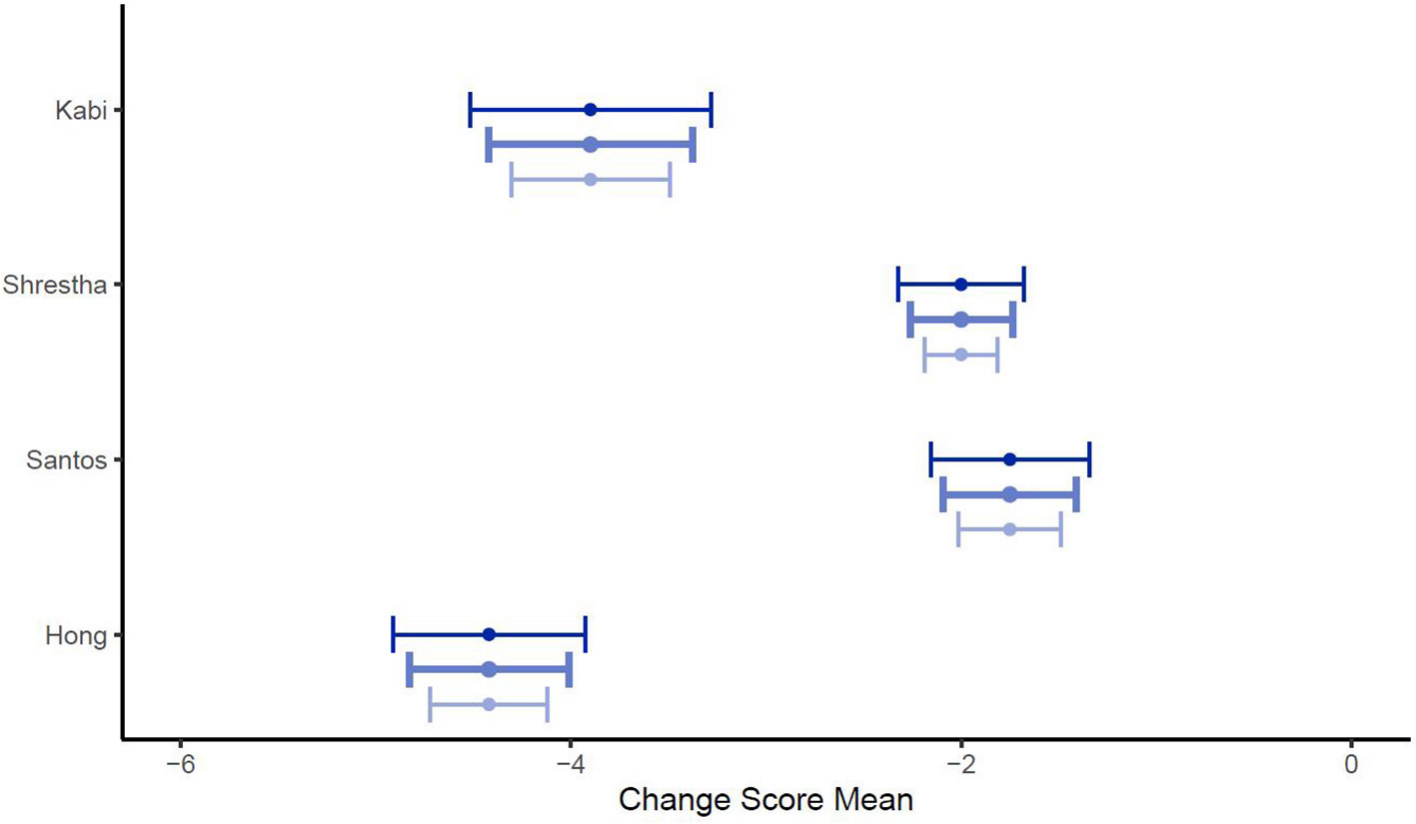
Forest plot for change scores of knowledge (*n* = 4) for ρ = (0.25, 0.5, 0.75) to calculate the standard deviation of the change score. Medium blue indicates ρ = 0.5, dark blue stands for ρ = 0.25 and light blue for ρ = 0.75.

Figures 11, 12 show the mean of the change scores and the estimated confidence intervals, setting ρ to {0.25, 0.5, 0.75} for both outcome variables separately. It can be seen that the smaller the ρ, the larger the confidence interval, as the standard deviation of a change score decreases with an increasing correlation. The smaller the ρ, the smaller the probability of obtaining a significant result, as the SD decreases with increasing ρ.


$$\begin{array}{l} SD_{change}=\sqrt{SD_{\;pre-sim}^{2}+\;SD_{\;post-sim}^{2}\;-\;(2\;*\rho^{*}SD_{pre-sim}\;*SD_{post-sim})} \end{array}$$

For the different given correlations, ρ, the confidence interval does not intersect zero. Therefore, it can be stated that the choice of a correlation ρ ε [0.25, 0.75] does not alter the results.

**Figure 13:**
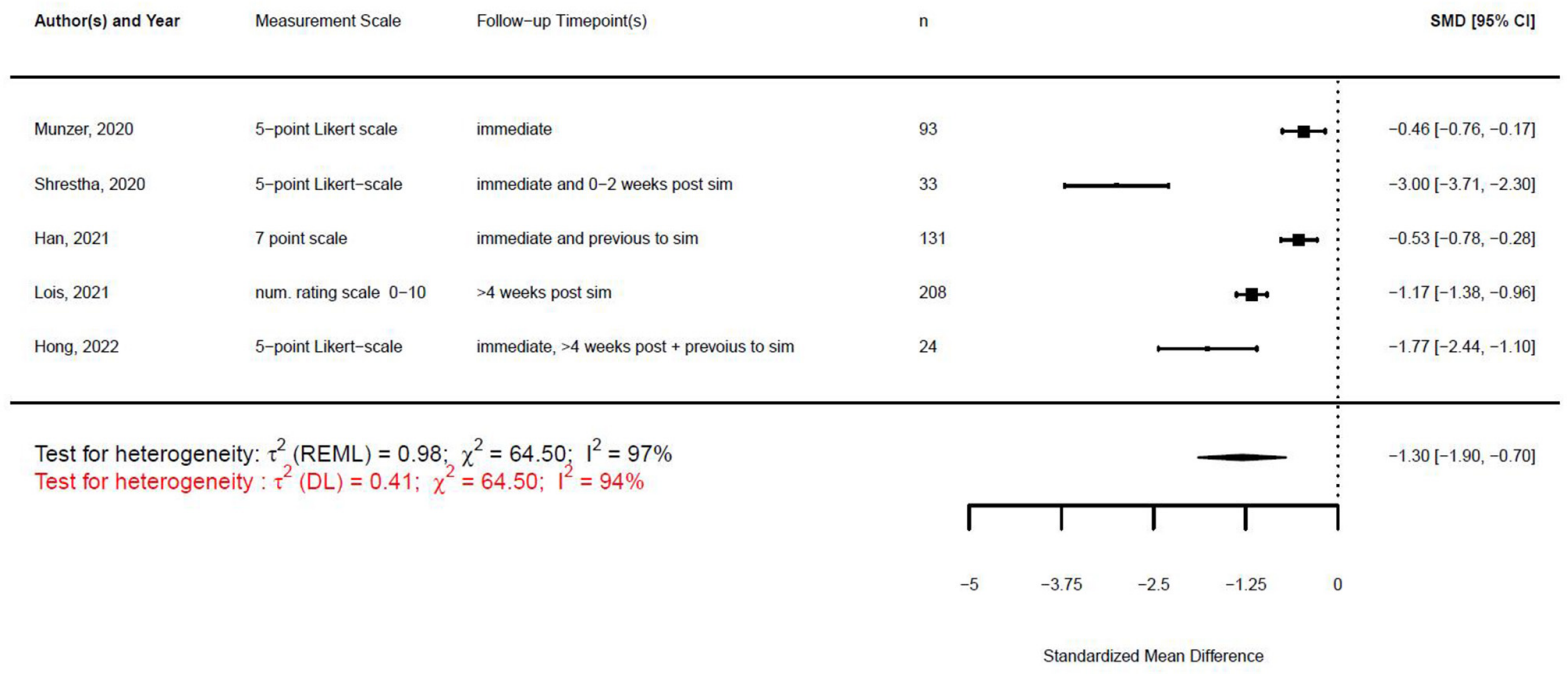
Forest plot for standardized mean difference of confidence (*n* = 5) with REML and DL estimator to compute heterogeneity.

**Figure 14:**
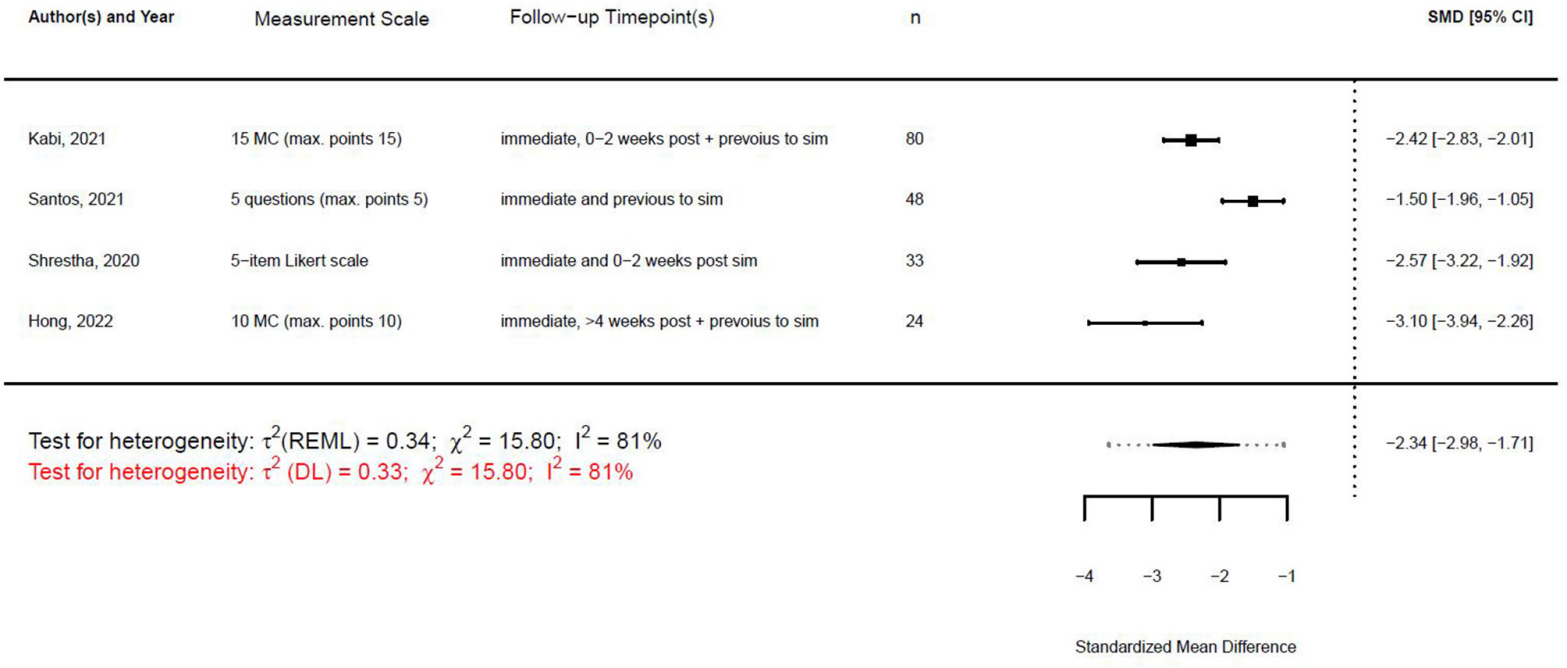
Forest plot for standardized mean difference of knowledge (*n* = 4) with REML and DL estimator to compute heterogeneity.

τ^2^ can be estimated using different methods. Applying both the restricted maximum likelihood (REML) estimator and the DL estimator led to similar estimates for τ^2^ in the random effects models for the SMD for knowledge. The estimates for τ^2^ confidence differed. However, the estimates for *I* ^2^ were similar. Figures 13, 14 show the forest plot for the models using the REML estimator, along with the results from the DL approach (highlighted in red).

### 3.7 Reporting biases

**Figure 15:**
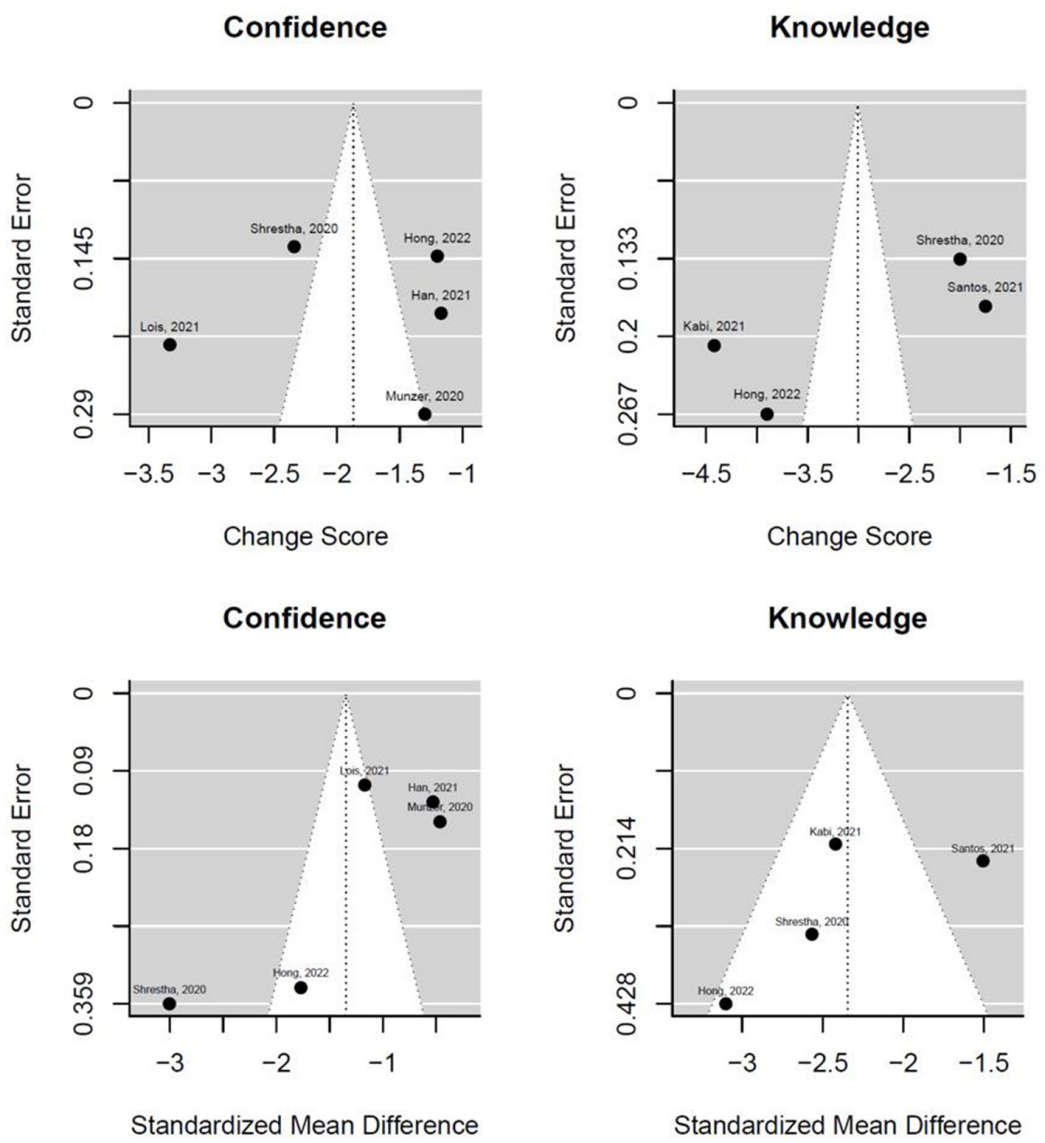
Funnel plots for the change score models and the standardized mean difference of confidence and knowledge.

Figure 15 shows the funnel plots for the change score models and the SMD models. The white funnel plot illustrates the 95% confidence interval around the effect estimate. It is notable that many studies fall outside the funnel plot. Moreover, the studies are distributed asymmetrically in the funnel plot, indicating publication bias.

### 3.8 Certainty of evidence

The certainty of evidence across the included studies varied. While most studies demonstrated improvements in confidence and knowledge post-simulation, the heterogeneity of the study designs, varying sample sizes, and methodological limitations reduced the overall confidence in the findings. In addition, the presence of publication bias, as indicated by asymmetrical funnel plots, further impacts the reliability of the results. Future studies with standardized methodologies and rigorous reporting are necessary to enhance the certainty of evidence in this area.

## 4 Discussion

This meta-analysis underscores the pivotal role of simulation-based training in preparing HCPs for effective and safe airway management during the COVID-19 pandemic. The findings reveal significant improvements in both confidence and knowledge, indicating that simulation training serves as a critical tool for enhancing technical and psychological readiness in high-stakes situations. These results are particularly relevant given the unprecedented challenges healthcare systems faced during the pandemic, where swift adaptation to new protocols was crucial for ensuring patient safety.

The observed improvements in confidence are noteworthy, as they extend beyond technical competence to address psychological preparedness. In high-pressure scenarios, such as managing critically ill COVID-19 patients, enhanced confidence is directly linked to improved clinical performance and decision-making. This finding aligns with previous research emphasizing the importance of building self-efficacy through experiential learning (24–26). Similarly, the substantial gains in knowledge highlight the capacity of simulation to reinforce key concepts, protocols, and procedures, translating into better clinical preparedness and patient care outcomes.

The systematic approach adopted in this review is a significant strength, offering a robust synthesis of data across diverse studies. By focusing on standardized mean differences (SMDs) and pre–post change scores, the analysis provides quantitative evidence of the effectiveness of simulation training. The application of the Kirkpatrick model further validated these findings, demonstrating that simulation training achieves measurable outcomes across multiple levels, from learning and confidence to clinical preparedness.

However, this review also highlights several limitations that must be considered. Heterogeneity among the included studies, as indicated by the *I*^2^ statistics, suggested variability in simulation design, implementation, and evaluation. The observed heterogeneity across the studies may be explained by the differences in participant populations (e.g., physicians, nurses, mixed teams), the type and intensity of simulation modalities (procedural vs. *in-situ* training), and the outcome measures assessed (knowledge, confidence, infection prevention). Clinically, such variation reflects the diversity of training contexts within real-world hospital systems and underlines the need for flexible implementation strategies tailored to local needs. While the use of a random effects model partially mitigated this issue, the findings must be interpreted within the context of these methodological differences.

Moreover, the presence of publication bias, as suggested by asymmetric funnel plots, presents a significant limitation. The underreporting of studies with neutral or negative outcomes may have skewed the overall results, potentially overestimating the effectiveness of simulation training. The sensitivity analyses provided some reassurance regarding the robustness of the findings, but the possibility of bias remains a concern.

To address these limitations, future research should prioritize methodological standardization and transparency. Large-scale, multicenter studies employing consistent evaluation metrics are needed to enhance the reliability and generalizability of the findings. Furthermore, efforts to reduce publication bias, such as encouraging the publication of studies with inconclusive or negative outcomes, will contribute to a more balanced understanding of the effectiveness of simulation-based training.

In addition to improvements in knowledge and confidence, several included studies also reported tangible clinical and system-level outcomes. For example, Buléon et al. (27) observed that healthcare providers who received large-scale simulation training were four times less likely to contract COVID-19 compared to untrained colleagues. Similarly, Delamarre et al. (28) demonstrated that a mass *in-situ* training program was associated with stable sick leave rates during the pandemic, suggesting improved system resilience. Beyond infection control, simulation also facilitated the identification and mitigation of latent safety threats: Shrestha et al. (29) reported issues such as missing medications and inadequate sample delivery mechanisms, while Lakissian et al. (30) highlighted challenges with donning and doffing PPE and team coordination during protected intubation. Addressing such threats through iterative simulation led to operational changes that directly supported patient and staff safety.

In conclusion, this review highlights the transformative potential of simulation training in pandemic preparedness. Beyond equipping healthcare providers with technical skills, simulation fosters psychological resilience and readiness, essential for effective performance in high-pressure environments. By addressing current limitations and advancing the evidence base, future research can further refine simulation training strategies, optimizing their impact in diverse healthcare settings.

## 5 Conclusion

Simulation-based training for airway management during the COVID-19 pandemic not only enhanced provider knowledge and confidence but also contributed to infection control, identification of latent safety threats, and improved system resilience. Simulation should therefore be considered an integral component of routine medical education and preparedness planning beyond the pandemic. Despite the limitations related to heterogeneity and potential publication bias, the overall findings suggest that such training significantly improves both confidence and knowledge among healthcare workers. Future research should focus on standardizing simulation protocols and exploring the long-term impact of these training interventions on patient outcomes.

## Data Availability

The original contributions presented in the study are included in the article/Supplementary material, further inquiries can be directed to the corresponding author.
