# Supplementary material for: Efficacy of simulation-based training for airway management in preparing hospitals for the COVID-19 pandemic: a systematic review
Source: Front Med (Lausanne). 2025 Dec 9;12:1656737. doi: 10.3389/fmed.2025.1656737 (PMC12723694; doi:10.3389/fmed.2025.1656737)
Supplement: Supplementary file 1 [file Data_Sheet_1.docx]

Supplemental Digital Content 1 – Search algorithms

| Pubmed | Methodology | (simulation [Title/Abstract]) AND ((airway management [Title/Abstract]) OR (intubation [Title/Abstract])) AND ((COVID [Title/Abstract]) OR (corona [Title/Abstract]) OR (SARS-COV2 [Title/Abstract])) NOT (box [Title/Abstract]) |
| --- | --- | --- |
|  | Results | 76 |
| Embase | Methodology | simulation:ab,ti AND (‘airway management’:ab,ti OR intubation:ab,ti) AND (COVID:ab,ti OR corona:ab,ti OR SARS-COV2:ab,ti) NOT box:ab,ti |
|  | Results | 51 (+49 duplicates with Pubmed) |
| Medline | Methodology | ((Simulation and (airway management or intubation) and (COVID or corona or SARS-COV2)) not box).ab,ti. |
|  | Results | 61 duplicates with Pubmed, 1 duplicate with Embase |
| Cochrane Library | Methodology | ((Simulation) AND (airway management OR intubation) AND (COVID OR corona OR SARS-COV2)) NOT box in Title Abstract Keyword - (Word variations have been searched) |
|  | Results | 14 (+ 6 duplicates with Pubmed) |
