# Supplementary material for: Efficacy of simulation-based training for airway management in preparing hospitals for the COVID-19 pandemic: a systematic review
Source: Front Med (Lausanne). 2025 Dec 9;12:1656737. doi: 10.3389/fmed.2025.1656737 (PMC12723694; doi:10.3389/fmed.2025.1656737)
Supplement: Supplementary file 2 [file Data_Sheet_2.docx]

Studies included in review

(n = 19)

Reports assessed for eligibility (n = 32)

Reports excluded:

- Not matching inclusion criteria (n = 3)
- Conference abstracts / posters / letter to the editor (n = 10)

Reports not retrieved

(n = 0)

Reports sought for retrieval

(n = 32)

Records screened

(n = 141)

Records excluded

(n = 109, not matching inclusion criteria)

**Included**

Total Studies included in review

(n = 20)

Studies included from reference lists of included publications

(n = 1)

**Screening**

**Identification**

**Identification of studies via databases according to the PRISMA guidelines**

Records removed before screening:

Duplicate records removed

(n = 117)

Records identified from:

PubMed (n = 76)

Embase (n = 100)

MedLine (n = 62)

Cochrane Library (n = 20)

Total (n = 258)
